# *catena*-Poly[[[dichloridomercury(II)]-μ-1,4-bis­(3-pyridyl­amino­meth­yl)benzene-κ^2^ *N*:*N*′] *N*,*N*-dimethyl­formamide monosolvate]

**DOI:** 10.1107/S1600536811026043

**Published:** 2011-07-09

**Authors:** Shan Gao, Seik Weng Ng

**Affiliations:** a Key Laboratory of Functional Inorganic Material Chemistry, Ministry of Education, Heilongjiang University, Harbin 150080, People’s Republic of China; b Department of Chemistry, University of Malaya, 50603 Kuala Lumpur, Malaysia; c Chemistry Department, Faculty of Science, King Abdulaziz University, PO Box 80203 Jeddah, Saudi Arabia

## Abstract

The crystal structure of the polymeric title compound, {[HgCl_2_(C_18_H_18_N_4_)]·C_3_H_7_NO}_*n*_, features an *N*-heterocyclic ligand which links adjacent HgCl_2_ units into a helical chain running along the *b* axis. The coordination geometry of the Hg^II^ atom is a distorted tetra­hedron. The dimethyl­formamide mol­ecule is disordered over two positions in a 1:1 ratio, and is linked to the complex mol­ecules *via* N—H⋯O hydrogen bonds.

## Related literature

For the structure of the *N*-heterocyclic ligand, see: Zhu *et al.* (2007 ▶).
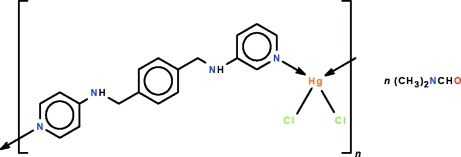


## Experimental

### 

#### Crystal data

[HgCl_2_(C_18_H_18_N_4_)]·C_3_H_7_NO
*M* *_r_* = 634.95
Monoclinic, 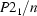

*a* = 8.4851 (9) Å
*b* = 15.1215 (14) Å
*c* = 19.490 (2) Å
β = 103.826 (2)°
*V* = 2428.2 (4) Å^3^
*Z* = 4
Mo *K*α radiation
μ = 6.58 mm^−1^
*T* = 293 K
0.15 × 0.11 × 0.11 mm

#### Data collection

Rigaku R-AXIS RAPID IP diffractometer
Absorption correction: multi-scan (*ABSCOR*; Higashi, 1995 ▶) *T*_min_ = 0.439, *T*_max_ = 0.531
22980 measured reflections
5479 independent reflections
2593 reflections with *I* > 2σ(*I*)
*R*_int_ = 0.089

#### Refinement

*R*[*F* ^2^ > 2σ(*F* ^2^)] = 0.051
*wR*(*F* ^2^) = 0.167
*S* = 1.05
5479 reflections
290 parameters
42 restraints
H-atom parameters constrained
Δρ_max_ = 1.26 e Å^−3^
Δρ_min_ = −1.31 e Å^−3^

### 

Data collection: *RAPID-AUTO* (Rigaku, 1998 ▶); cell refinement: *RAPID-AUTO*; data reduction: *CrystalClear* (Rigaku/MSC, 2002 ▶); program(s) used to solve structure: *SHELXS97* (Sheldrick, 2008 ▶); program(s) used to refine structure: *SHELXL97* (Sheldrick, 2008 ▶); molecular graphics: *X-SEED* (Barbour, 2001 ▶); software used to prepare material for publication: *publCIF* (Westrip, 2010 ▶).

## Supplementary Material

Crystal structure: contains datablock(s) global, I. DOI: 10.1107/S1600536811026043/xu5257sup1.cif

Structure factors: contains datablock(s) I. DOI: 10.1107/S1600536811026043/xu5257Isup2.hkl

Additional supplementary materials:  crystallographic information; 3D view; checkCIF report

## Figures and Tables

**Table 1:** Selected bond lengths (Å)

Hg1—N1	2.395 (7)
Hg1—N4^i^	2.308 (6)
Hg1—Cl1	2.355 (3)
Hg1—Cl2	2.391 (3)

Symmetry code: (i) 
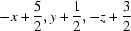
.

**Table 2:** Hydrogen-bond geometry (Å, °)

*D*—H⋯*A*	*D*—H	H⋯*A*	*D*⋯*A*	*D*—H⋯*A*
N2—H2*N*⋯O1	0.88	2.15	3.03 (3)	174
N2—H2*N*⋯O1′	0.88	2.11	2.99 (3)	180
N3—H3*N*⋯O1^ii^	0.88	2.14	3.01 (3)	166
N3—H3*N*⋯O1′^ii^	0.88	2.13	2.98 (3)	162

Symmetry code: (ii) 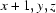.

**Fig. 1.**
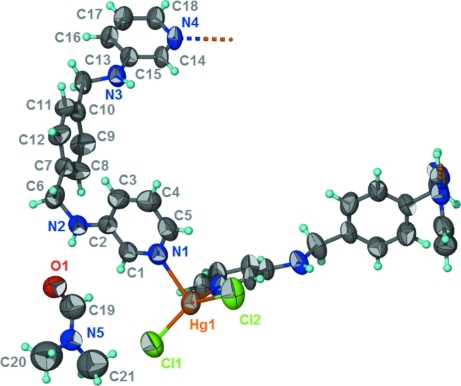
Thermal ellipsoid plot (Barbour, 2001) of a portion of the polymeric chain structure of HgCl2(C18H18N4).DMF at the 50% probability level; hydrogen atoms are drawn as spheres of arbitrary. radius.

## supplementary crystallographic information

### Comment

1,4-Bis(2-pyridylaminomethyl)benzene is a flexible *N*-heterocycle whose pyridyl and amino N-atoms are capable for forming coordination polymers (Zhu *et al.*, 2007). The crystal structure of HgCl_2_(C_18_H_18_N_4_)^.^DMF features the *N*-heterocycle linking adjacent HgCl_2_ units into a helical chain (Scheme I, Fig. 1). The geometry of Hg^II^ is a tetrahedron. The lattice DMF molecule is disordered in two positions in a 1:1 ratio. The *N*-heterocycle forms an N–H···O hydrogen bond to the solvent molecule at an N···O distance of 2.99 (3) and 3.03 (3) Å; the hydrogen bond probably stabilizes the solvent molecule so that it is not lost during crystallization.

### Experimental

A THF solution (10 ml) of mercuric chloride (2 mmol) was mixed with a DMF solution (5 ml) of 1,4-bis(3-pyridylaminomethyl)benzene (2 mmol). The solution was filtered and sent aside for the grown of colorless crystals.

### Refinement

Carbon-bound H-atoms were placed in calculated positions (C–H 0.93 Å) and were included in the refinement in the riding model approximation, with *U*(H) set to 1.2*U*(C). The amino H-atoms similar treated (N–H 0.86 Å).

The lattice DMF molecule is disordered over two sites; the disorder could not be refined, and was assumed to be a 1:1 type of disorder. The C–O distances were restrained to 1.25±0.01 Å, the C_carbonyl_–N distances to 1.35±0.01 Å and the *N*–C_methyl_ distances to 1.45±0.01 Å. Each component was retrained to planar, with a maximum deviation of 0.01 Å. The temperature factors of the primed atoms were set to those of the unprimed ones, and the anisotropic temperature factors were restrained to be nearly isotropic.

The final difference Fourier map had peaks/holes in the vicinity of Hg1.

### Crystal data

**Table:**

[HgCl_2_(C_18_H_18_N_4_)]·C_3_H_7_NO	*F*(000) = 1232
*M**_r_* = 634.95	*D*_x_ = 1.737 Mg m^−^^3^
Monoclinic, *P*2_1_/*n*	Mo *K*α radiation, λ = 0.71073 Å
Hall symbol: -P 2yn	Cell parameters from 10259 reflections
*a* = 8.4851 (9) Å	θ = 3.2–27.5°
*b* = 15.1215 (14) Å	µ = 6.58 mm^−^^1^
*c* = 19.490 (2) Å	*T* = 293 K
β = 103.826 (2)°	Prism, colorless
*V* = 2428.2 (4) Å^3^	0.15 × 0.11 × 0.11 mm
*Z* = 4	

### Data collection

**Table:**

Rigaku R-AXIS RAPID IP diffractometer	5479 independent reflections
Radiation source: fine-focus sealed tube	2593 reflections with *I* > 2σ(*I*)
graphite	*R*_int_ = 0.089
ω scan	θ_max_ = 27.5°, θ_min_ = 3.2°
Absorption correction: multi-scan (*ABSCOR*; Higashi, 1995)	*h* = −11→11
*T*_min_ = 0.439, *T*_max_ = 0.531	*k* = −19→19
22980 measured reflections	*l* = −25→22

### Refinement

**Table:**

Refinement on *F*^2^	Primary atom site location: structure-invariant direct methods
Least-squares matrix: full	Secondary atom site location: difference Fourier map
*R*[*F*^2^ > 2σ(*F*^2^)] = 0.051	Hydrogen site location: inferred from neighbouring sites
*wR*(*F*^2^) = 0.167	H-atom parameters constrained
*S* = 1.05	*w* = 1/[σ^2^(*F*_o_^2^) + (0.0656*P*)^2^] where *P* = (*F*_o_^2^ + 2*F*_c_^2^)/3
5479 reflections	(Δ/σ)_max_ = 0.001
290 parameters	Δρ_max_ = 1.26 e Å^−^^3^
42 restraints	Δρ_min_ = −1.31 e Å^−^^3^

### Fractional atomic coordinates and isotropic or equivalent isotropic displacement parameters (Å^2^)

**Table:**

	*x*	*y*	*z*	*U*_iso_*/*U*_eq_	Occ. (<1)
Hg1	0.65054 (5)	0.63785 (2)	0.73300 (2)	0.0944 (2)	
Cl1	0.3880 (4)	0.5916 (3)	0.7371 (2)	0.1488 (12)	
Cl2	0.7868 (4)	0.77413 (15)	0.72364 (19)	0.1366 (11)	
N1	0.7052 (9)	0.5581 (4)	0.6351 (4)	0.0845 (19)	
N2	0.5616 (9)	0.3645 (4)	0.5347 (5)	0.097 (2)	
H2N	0.4912	0.3482	0.5589	0.116*	
N3	1.2859 (9)	0.1242 (4)	0.5624 (4)	0.089 (2)	
H3N	1.2888	0.1819	0.5685	0.106*	
N4	1.6587 (8)	0.0700 (4)	0.6783 (3)	0.0778 (18)	
C1	0.6262 (10)	0.4841 (5)	0.6124 (5)	0.077 (2)	
H1	0.5528	0.4619	0.6366	0.092*	
C2	0.6491 (10)	0.4383 (5)	0.5535 (5)	0.078 (2)	
C3	0.7569 (11)	0.4741 (7)	0.5156 (5)	0.097 (3)	
H3	0.7720	0.4457	0.4753	0.117*	
C4	0.8377 (13)	0.5496 (7)	0.5381 (6)	0.105 (3)	
H4	0.9108	0.5734	0.5144	0.126*	
C5	0.8089 (11)	0.5909 (6)	0.5976 (6)	0.098 (3)	
H5	0.8631	0.6435	0.6127	0.118*	
C6	0.5810 (12)	0.3099 (7)	0.4743 (5)	0.108 (3)	
H6A	0.5769	0.3485	0.4342	0.130*	
H6B	0.4894	0.2698	0.4617	0.130*	
C7	0.7345 (10)	0.2565 (5)	0.4868 (5)	0.077 (2)	
C8	0.7897 (12)	0.2164 (6)	0.5502 (6)	0.100 (3)	
H8	0.7379	0.2257	0.5866	0.120*	
C9	0.9204 (13)	0.1631 (7)	0.5601 (5)	0.101 (3)	
H9	0.9559	0.1349	0.6035	0.121*	
C10	1.0050 (10)	0.1482 (5)	0.5074 (5)	0.073 (2)	
C11	0.9536 (11)	0.1896 (6)	0.4447 (5)	0.085 (2)	
H11	1.0070	0.1811	0.4087	0.102*	
C12	0.8207 (12)	0.2447 (6)	0.4349 (5)	0.093 (3)	
H12	0.7875	0.2749	0.3923	0.111*	
C13	1.1469 (10)	0.0856 (6)	0.5190 (6)	0.098 (3)	
H13A	1.1204	0.0317	0.5408	0.118*	
H13B	1.1685	0.0701	0.4738	0.118*	
C14	1.5277 (9)	0.1115 (5)	0.6468 (4)	0.0675 (19)	
H14	1.5119	0.1688	0.6610	0.081*	
C15	1.4147 (9)	0.0755 (5)	0.5950 (4)	0.0700 (19)	
C16	1.4362 (11)	−0.0123 (5)	0.5778 (5)	0.083 (2)	
H16	1.3588	−0.0406	0.5429	0.100*	
C17	1.5701 (12)	−0.0560 (6)	0.6123 (5)	0.092 (3)	
H17	1.5835	−0.1152	0.6020	0.111*	
C18	1.6827 (11)	−0.0152 (5)	0.6607 (5)	0.083 (2)	
H18	1.7778	−0.0446	0.6825	0.100*	
O1	0.300 (4)	0.314 (2)	0.6094 (14)	0.110 (3)	0.50
N5	0.181 (3)	0.3560 (13)	0.7007 (11)	0.085 (3)	0.50
C19	0.303 (3)	0.3275 (14)	0.6732 (14)	0.128 (6)	0.50
H19	0.4011	0.3163	0.7053	0.154*	0.50
C20	0.025 (3)	0.375 (2)	0.6545 (18)	0.197 (11)	0.50
H20A	0.0333	0.4262	0.6267	0.295*	0.50
H20B	−0.0525	0.3856	0.6823	0.295*	0.50
H20C	−0.0096	0.3252	0.6239	0.295*	0.50
C21	0.180 (5)	0.380 (2)	0.7725 (13)	0.185 (9)	0.50
H21A	0.1230	0.4349	0.7723	0.278*	0.50
H21B	0.2899	0.3868	0.7998	0.278*	0.50
H21C	0.1273	0.3346	0.7931	0.278*	0.50
O1'	0.323 (4)	0.309 (2)	0.6172 (14)	0.110 (3)	0.50
N5'	0.181 (3)	0.3701 (11)	0.6889 (12)	0.085 (3)	0.50
C19'	0.200 (3)	0.3494 (13)	0.6242 (13)	0.128 (6)	0.50
H19'	0.1203	0.3654	0.5844	0.154*	0.50
C20'	0.052 (4)	0.416 (2)	0.712 (2)	0.197 (11)	0.50
H20D	−0.0339	0.3751	0.7128	0.295*	0.50
H20E	0.0113	0.4629	0.6793	0.295*	0.50
H20F	0.0940	0.4398	0.7580	0.295*	0.50
C21'	0.308 (3)	0.344 (2)	0.7492 (15)	0.185 (9)	0.50
H21D	0.3351	0.2828	0.7443	0.278*	0.50
H21E	0.2704	0.3509	0.7917	0.278*	0.50
H21F	0.4020	0.3798	0.7517	0.278*	0.50

### Atomic displacement parameters (Å^2^)

**Table:**

	*U*^11^	*U*^22^	*U*^33^	*U*^12^	*U*^13^	*U*^23^
Hg1	0.0921 (3)	0.0868 (3)	0.0909 (3)	0.00588 (18)	−0.0045 (2)	−0.00621 (18)
Cl1	0.096 (2)	0.202 (3)	0.142 (3)	−0.024 (2)	0.017 (2)	−0.030 (3)
Cl2	0.139 (3)	0.0719 (13)	0.172 (3)	0.0028 (14)	−0.015 (2)	0.0193 (16)
N1	0.071 (5)	0.083 (4)	0.092 (5)	0.005 (4)	0.007 (4)	−0.001 (4)
N2	0.065 (5)	0.099 (5)	0.123 (7)	0.002 (4)	0.014 (5)	−0.031 (5)
N3	0.066 (5)	0.072 (4)	0.116 (6)	0.005 (3)	−0.003 (4)	−0.016 (4)
N4	0.079 (5)	0.073 (4)	0.065 (4)	−0.009 (3)	−0.014 (3)	−0.005 (3)
C1	0.062 (5)	0.080 (5)	0.081 (5)	0.007 (4)	0.002 (4)	0.002 (4)
C2	0.062 (5)	0.078 (5)	0.089 (6)	0.013 (4)	0.010 (5)	−0.010 (5)
C3	0.078 (7)	0.114 (8)	0.097 (7)	0.020 (5)	0.014 (6)	−0.012 (6)
C4	0.107 (8)	0.103 (7)	0.113 (8)	0.004 (6)	0.041 (7)	0.005 (6)
C5	0.072 (6)	0.086 (6)	0.126 (8)	−0.002 (5)	0.004 (6)	0.003 (6)
C6	0.096 (7)	0.108 (7)	0.101 (7)	0.026 (6)	−0.016 (6)	−0.034 (6)
C7	0.064 (5)	0.081 (5)	0.082 (6)	0.013 (4)	0.005 (5)	−0.010 (4)
C8	0.092 (7)	0.113 (7)	0.101 (7)	0.036 (6)	0.037 (6)	0.020 (6)
C9	0.113 (8)	0.122 (7)	0.066 (6)	0.029 (6)	0.017 (6)	0.021 (5)
C10	0.057 (5)	0.070 (4)	0.080 (6)	0.007 (4)	−0.006 (4)	−0.007 (4)
C11	0.083 (6)	0.100 (6)	0.075 (6)	0.016 (5)	0.023 (5)	−0.007 (5)
C12	0.104 (8)	0.107 (6)	0.063 (5)	0.025 (5)	0.012 (5)	−0.002 (5)
C13	0.066 (6)	0.090 (6)	0.119 (8)	0.007 (5)	−0.017 (5)	−0.029 (5)
C14	0.058 (5)	0.068 (4)	0.056 (4)	0.010 (3)	−0.027 (3)	−0.004 (3)
C15	0.063 (5)	0.069 (4)	0.068 (5)	0.012 (4)	−0.004 (4)	0.003 (4)
C16	0.086 (6)	0.067 (4)	0.083 (6)	0.007 (4)	−0.006 (5)	−0.011 (4)
C17	0.094 (7)	0.070 (5)	0.099 (7)	0.009 (5)	−0.006 (6)	−0.006 (5)
C18	0.082 (6)	0.070 (5)	0.083 (6)	0.007 (4)	−0.009 (5)	−0.003 (4)
O1	0.108 (7)	0.101 (4)	0.125 (6)	0.006 (5)	0.035 (5)	−0.011 (4)
N5	0.083 (5)	0.087 (6)	0.082 (6)	0.010 (4)	0.015 (5)	0.016 (5)
C19	0.125 (10)	0.134 (9)	0.122 (10)	0.015 (7)	0.023 (8)	0.009 (8)
C20	0.188 (13)	0.210 (14)	0.193 (14)	0.023 (9)	0.047 (10)	−0.009 (9)
C21	0.189 (12)	0.195 (12)	0.177 (12)	0.005 (9)	0.054 (9)	0.000 (9)
O1'	0.108 (7)	0.101 (4)	0.125 (6)	0.006 (5)	0.035 (5)	−0.011 (4)
N5'	0.083 (5)	0.087 (6)	0.082 (6)	0.010 (4)	0.015 (5)	0.016 (5)
C19'	0.125 (10)	0.134 (9)	0.122 (10)	0.015 (7)	0.023 (8)	0.009 (8)
C20'	0.188 (13)	0.210 (14)	0.193 (14)	0.023 (9)	0.047 (10)	−0.009 (9)
C21'	0.189 (12)	0.195 (12)	0.177 (12)	0.005 (9)	0.054 (9)	0.000 (9)

### Geometric parameters (Å, °)

**Table:**

Hg1—N1	2.395 (7)	C11—H11	0.9300
Hg1—N4^i^	2.308 (6)	C12—H12	0.9300
Hg1—Cl1	2.355 (3)	C13—H13A	0.9700
Hg1—Cl2	2.391 (3)	C13—H13B	0.9700
N1—C1	1.325 (10)	C14—C15	1.331 (10)
N1—C5	1.364 (11)	C14—H14	0.9300
N2—C2	1.342 (10)	C15—C16	1.392 (10)
N2—C6	1.479 (11)	C16—C17	1.346 (12)
N2—H2N	0.8800	C16—H16	0.9300
N3—C15	1.345 (10)	C17—C18	1.324 (11)
N3—C13	1.404 (10)	C17—H17	0.9300
N3—H3N	0.8800	C18—H18	0.9300
N4—C14	1.296 (9)	O1—C19	1.253 (10)
N4—C18	1.361 (9)	N5—C19	1.344 (10)
N4—Hg1^ii^	2.308 (6)	N5—C20	1.440 (10)
C1—C2	1.392 (11)	N5—C21	1.448 (10)
C1—H1	0.9300	C19—H19	0.9300
C2—C3	1.414 (12)	C20—H20A	0.9600
C3—C4	1.351 (13)	C20—H20B	0.9600
C3—H3	0.9300	C20—H20C	0.9600
C4—C5	1.390 (13)	C21—H21A	0.9600
C4—H4	0.9300	C21—H21B	0.9600
C5—H5	0.9300	C21—H21C	0.9600
C6—C7	1.502 (11)	O1'—C19'	1.250 (10)
C6—H6A	0.9700	N5'—C19'	1.346 (10)
C6—H6B	0.9700	N5'—C20'	1.448 (10)
C7—C8	1.356 (12)	N5'—C21'	1.449 (10)
C7—C12	1.394 (11)	C19'—H19'	0.9300
C8—C9	1.347 (12)	C20'—H20D	0.9600
C8—H8	0.9300	C20'—H20E	0.9600
C9—C10	1.405 (12)	C20'—H20F	0.9600
C9—H9	0.9300	C21'—H21D	0.9600
C10—C11	1.348 (11)	C21'—H21E	0.9600
C10—C13	1.506 (11)	C21'—H21F	0.9600
C11—C12	1.378 (11)		
			
N4^i^—Hg1—Cl1	110.0 (2)	C10—C11—C12	118.9 (8)
N4^i^—Hg1—Cl2	100.07 (18)	C10—C11—H11	120.6
Cl1—Hg1—Cl2	137.49 (12)	C12—C11—H11	120.6
N4^i^—Hg1—N1	97.9 (2)	C11—C12—C7	122.1 (8)
Cl1—Hg1—N1	104.0 (2)	C11—C12—H12	119.0
Cl2—Hg1—N1	100.6 (2)	C7—C12—H12	119.0
C1—N1—C5	117.9 (8)	N3—C13—C10	110.8 (7)
C1—N1—Hg1	120.9 (6)	N3—C13—H13A	109.5
C5—N1—Hg1	121.1 (6)	C10—C13—H13A	109.5
C2—N2—C6	121.5 (8)	N3—C13—H13B	109.5
C2—N2—H2N	119.2	C10—C13—H13B	109.5
C6—N2—H2N	119.2	H13A—C13—H13B	108.1
C15—N3—C13	121.9 (7)	N4—C14—C15	122.8 (7)
C15—N3—H3N	119.0	N4—C14—H14	118.6
C13—N3—H3N	119.0	C15—C14—H14	118.6
C14—N4—C18	120.1 (7)	C14—C15—N3	119.4 (7)
C14—N4—Hg1^ii^	120.6 (5)	C14—C15—C16	117.4 (7)
C18—N4—Hg1^ii^	119.3 (5)	N3—C15—C16	123.2 (8)
N1—C1—C2	122.4 (8)	C17—C16—C15	119.4 (8)
N1—C1—H1	118.8	C17—C16—H16	120.3
C2—C1—H1	118.8	C15—C16—H16	120.3
N2—C2—C1	117.5 (8)	C18—C17—C16	120.4 (8)
N2—C2—C3	124.0 (8)	C18—C17—H17	119.8
C1—C2—C3	118.4 (8)	C16—C17—H17	119.8
C4—C3—C2	119.7 (9)	C17—C18—N4	119.7 (8)
C4—C3—H3	120.1	C17—C18—H18	120.2
C2—C3—H3	120.1	N4—C18—H18	120.2
C3—C4—C5	118.3 (9)	C19—N5—C20	120 (3)
C3—C4—H4	120.9	C19—N5—C21	130 (3)
C5—C4—H4	120.9	C20—N5—C21	110 (3)
N1—C5—C4	123.3 (9)	O1—C19—N5	128 (3)
N1—C5—H5	118.4	O1—C19—H19	116.2
C4—C5—H5	118.4	N5—C19—H19	116.2
N2—C6—C7	115.3 (8)	C19'—N5'—C20'	132 (3)
N2—C6—H6A	108.4	C19'—N5'—C21'	118 (2)
C7—C6—H6A	108.4	C20'—N5'—C21'	111 (2)
N2—C6—H6B	108.4	O1'—C19'—N5'	121 (3)
C7—C6—H6B	108.4	O1'—C19'—H19'	119.7
H6A—C6—H6B	107.5	N5'—C19'—H19'	119.7
C8—C7—C12	118.5 (8)	N5'—C20'—H20D	109.5
C8—C7—C6	119.1 (8)	N5'—C20'—H20E	109.5
C12—C7—C6	122.4 (9)	H20D—C20'—H20E	109.5
C9—C8—C7	119.4 (8)	N5'—C20'—H20F	109.5
C9—C8—H8	120.3	H20D—C20'—H20F	109.5
C7—C8—H8	120.3	H20E—C20'—H20F	109.5
C8—C9—C10	122.6 (9)	N5'—C21'—H21D	109.5
C8—C9—H9	118.7	N5'—C21'—H21E	109.5
C10—C9—H9	118.7	H21D—C21'—H21E	109.5
C11—C10—C9	118.5 (7)	N5'—C21'—H21F	109.5
C11—C10—C13	120.1 (8)	H21D—C21'—H21F	109.5
C9—C10—C13	121.4 (8)	H21E—C21'—H21F	109.5
			
N4^i^—Hg1—N1—C1	90.8 (6)	C8—C9—C10—C13	−177.7 (10)
Cl1—Hg1—N1—C1	−22.2 (7)	C9—C10—C11—C12	0.1 (13)
Cl2—Hg1—N1—C1	−167.3 (6)	C13—C10—C11—C12	178.2 (8)
N4^i^—Hg1—N1—C5	−94.4 (7)	C10—C11—C12—C7	−2.3 (15)
Cl1—Hg1—N1—C5	152.6 (6)	C8—C7—C12—C11	4.0 (15)
Cl2—Hg1—N1—C5	7.5 (7)	C6—C7—C12—C11	−174.4 (9)
C5—N1—C1—C2	1.8 (12)	C15—N3—C13—C10	162.7 (8)
Hg1—N1—C1—C2	176.8 (6)	C11—C10—C13—N3	105.7 (10)
C6—N2—C2—C1	−178.2 (7)	C9—C10—C13—N3	−76.3 (12)
C6—N2—C2—C3	4.8 (14)	C18—N4—C14—C15	−2.5 (12)
N1—C1—C2—N2	−179.4 (8)	Hg1^ii^—N4—C14—C15	179.4 (6)
N1—C1—C2—C3	−2.1 (13)	N4—C14—C15—N3	−176.6 (8)
N2—C2—C3—C4	178.9 (9)	N4—C14—C15—C16	4.0 (12)
C1—C2—C3—C4	1.8 (14)	C13—N3—C15—C14	−165.7 (8)
C2—C3—C4—C5	−1.4 (15)	C13—N3—C15—C16	13.6 (14)
C1—N1—C5—C4	−1.3 (14)	C14—C15—C16—C17	−1.8 (13)
Hg1—N1—C5—C4	−176.3 (7)	N3—C15—C16—C17	178.9 (9)
C3—C4—C5—N1	1.1 (16)	C15—C16—C17—C18	−1.9 (15)
C2—N2—C6—C7	73.2 (12)	C16—C17—C18—N4	3.5 (14)
N2—C6—C7—C8	40.2 (13)	C14—N4—C18—C17	−1.4 (12)
N2—C6—C7—C12	−141.5 (9)	Hg1^ii^—N4—C18—C17	176.7 (7)
C12—C7—C8—C9	−3.4 (15)	C20—N5—C19—O1	0.0 (2)
C6—C7—C8—C9	175.0 (10)	C21—N5—C19—O1	−174 (3)
C7—C8—C9—C10	1.3 (17)	C20'—N5'—C19'—O1'	180.0 (4)
C8—C9—C10—C11	0.4 (15)	C21'—N5'—C19'—O1'	0.0 (3)

Symmetry codes: (i) −*x*+5/2, *y*+1/2, −*z*+3/2; (ii) −*x*+5/2, *y*−1/2, −*z*+3/2.

### Hydrogen-bond geometry (Å, °)

**Table:**

*D*—H···*A*	*D*—H	H···*A*	*D*···*A*	*D*—H···*A*
N2—H2N···O1	0.88	2.15	3.03 (3)	174
N2—H2N···O1'	0.88	2.11	2.99 (3)	180
N3—H3N···O1^iii^	0.88	2.14	3.01 (3)	166
N3—H3N···O1'^iii^	0.88	2.13	2.98 (3)	162

Symmetry codes: (iii) *x*+1, *y*, *z*.

## References

[bb1] Barbour, L. J. (2001). *J. Supramol. Chem.* **1**, 189–191.

[bb2] Higashi, T. (1995). *ABSCOR* Rigaku Corporation, Tokyo, Japan.

[bb3] Rigaku (1998). *RAPID-AUTO* Rigaku Corporation, Tokyo, Japan.

[bb4] Rigaku/MSC (2002). *CrystalClear* Rigaku/MSC Inc., The Woodlands, Texas, USA.

[bb5] Sheldrick, G. M. (2008). *Acta Cryst.* A**64**, 112–122. 10.1107/S0108767307043930 18156677

[bb6] Westrip, S. P. (2010). *J. Appl. Cryst.* **43**, 920–925.

[bb7] Zhu, L.-N., Gao, S. & Huo, L.-H. (2007). *Acta Cryst.* E**63**, o4459.

